# Oral Mycobiome Differences in Various Spatial Niches With and Without Severe Early Childhood Caries

**DOI:** 10.3389/fped.2021.748656

**Published:** 2021-11-15

**Authors:** Yuqi Cui, Yinuo Wang, Yuwen Zhang, Liangyue Pang, Yan Zhou, Huancai Lin, Ye Tao

**Affiliations:** ^1^Department of Preventive Dentistry, Hospital of Stomatology, Sun Yat-Sen University, Guangzhou, China; ^2^Guangdong Provincial Key Laboratory of Stomatology, Guanghua School of Stomatology, Sun Yat-Sen University, Guangzhou, China

**Keywords:** ITS2, dental plaque, saliva, mycobiome, S-ECC

## Abstract

**Purpose:** Severe early childhood caries (S-ECC) is a microbe-mediated disease with tooth hard tissue destruction. However, the role of the fungal community in various ecological niches of deciduous dental caries has not been fully elucidated. This study aimed to analyze the differences of mycobiome in diverse spatial niches with and without S-ECC.

**Method:** A total of 48 samples were obtained from 8 S-ECC children (SE group) and 8 caries-free children (CF group) aged 4–5 years. Unstimulated saliva (S), healthy supragingival plaque (FMIX), mixed plaque from decayed teeth (SMIX) and carious lesion (DMIX) samples were collected. The ITS2 region of the fungi was amplified and sequenced using the Ion S5™XL platform.

**Results:** A total of 281 species were identified. *Candida albicans* showed relatively higher abundance in S-ECC children, while *Alternaria alternata* and *Bipolaris sorokiniana* were more enriched in CF group. In this study, the relative abundance of *C. albicans* in CF.FMIX (0.4%), SE.FMIX (12.5%), SE.SMIX (24.0%), and SE.DMIX (37.2%) increased successively. Significant differences of fungal species richness and diversity were observed between SE.FMIX-SE.SMIX, SE.FMIX-SE.DMIX (*P* < 0.05).

**Conclusions:** The diversity of fungal communities in S-ECC children showed significant differences in various spatial niches of primary teeth. The richness of *C. albicans* was closely related to the caries states and depth, suggesting that it may play a crucial role in caries pathogenicity.

## Introduction

Early childhood caries (ECC) is one of the most common chronic infectious diseases in children (1). Severe ECC (S-ECC), which occurs at an earlier age, with characteristics of rapid development, wide invasion and seriously endangering children's oral and systemic health (2). In developed countries, such as United States, the prevalence of primary dental caries among pre-school children was 22.7% (3), and the average cost for total dental rehabilitation (including restorations, pulpotomies, stainless steel crowns, and extractions) was $7,303 per case (4). In China, according to the results of the Fourth National Oral Epidemiological Survey, 71.9% children aged 5 years experienced ECC, while the treatment rate was only 4.1% (5). Hence, it is necessary to strengthen understanding of S-ECC pathogenesis to enhance the prevention and early intervention.

The etiological factor of S-ECC is closely related to the dysbiosis of oral microbial community (6). Seeking for microbiological markers related to caries risk is the key to achieve ecological prevention. Fungal microbiome (mycobiome) is an essential component of oral microbiota. Despite its low abundance, the mycobiome has a significant impact on human health and disease (7). Recent application of the next-generation sequencing technology in microbiome research has greatly expanded the understanding of oral fungi. Currently, more than 100 fungal species have been identified to exist in the oral cavity (8). Sequencing research had consistently detected that fungal microbiome was related to oral diseases such as caries and periodontitis (9, 10).

Nevertheless, the characteristics of fungal community in the oral cavity have not been fully elucidated, especially in children with deciduous dentition. Most studies of oral fungi in children have focused on supragingival plaque of mixed dentition, and concluded: (1) ECC-related fungi include *Candida albicans, Cryptococcus neoformans, Candida sake* and *Nigrospora oryzae*; (2) The occurrence of ECC was associated to the decrease of fungal diversity (10–12). In particular, *C. albicans* was dominant in the oral fungal profile, and experimental studies have shown that it could produce caries in collaboration with *S. mutans* (13–16). A meta-analysis in 2018 including nine cross-sectional epidemiological studies found that children with detectable *C. albicans* had a five times higher risk of ECC than children who did not (17). However, there is no consensus about the association between *C. albicans* and dental caries. Jesus et al. reported no significantly difference in the abundance of *C. albicans* between S-ECC and caries-free children (*P* > 0.05) (18). Moreover, little attention has been paid to the spatial heterogeneity of fungal community in oral cavity.

This study aimed to elucidate mycobiome characteristics in each ecological nich in S-ECC and caries-free children. We performed next-generation sequencing to analyze the fungal composition in saliva and different spatial niches of teeth (including 6 positions), which can provide theoretical basis for the role of oral fungal profile in maintaining steady state of oral health and its relationship with S-ECC, and find caries risk microbial signs and screening caries susceptible population.

## Materials and Methods

### Population

This study was approved by the Ethics Committee of the Hospital of Stomatology, Sun Yat-sen University, in Guangzhou, China. Children aged 4–5 years with complete deciduous teeth were invited to participate in this study and informed consent was signed by their guardians. Exclusion criteria were as follows: (1) individuals who have systemic diseases and had used antibiotics within 3 months; (2) individuals who suffered from salivary gland disease and/or other oral diseases (such as periodontitis and oral mucosal disease); (3) application of topical and systemic fluoride within 6 months; (4) primary teeth with enamel hypoplasia (19). Children's general oral hygiene habits and dietary habits were investigated by questionnaires from their guardians or caregivers.

### Clinical Examination

The International Caries Detection and Assessment System II (ICDASII) criteria was adopted for clinical examination (20). Eight children with S-ECC [decayed, missing, or filled tooth surfaces (dmfs) ≥ 8] and 8 caries-free (dmfs = 0) children aged 49–63 months were participated in the study (21). Oral examinations were performed by an experienced dentist (Tao Y) using a standard mouth mirror, headlamp, and community periodontal index (CPI) probe.

### Sample Collection

Children were required to refrain from tooth brushing for 12 ± 4 h and avoid eating for 2 h before sampling. Unstimulated whole saliva from CF and SE children (CF.S and SE.S) was collected into a 15 ml sterile centrifuge tube by spitting method. Samples of supragingival plaque were collected respectively from tooth surfaces and categorized as follows: mixed supragingival plaque from available healthy tooth surfaces (FMIX) (12, 18) and mixed plaque from decayed tooth surfaces (SMIX) (22) were collected using a sterile cotton swab; carious tissues of all decayed teeth (DMIX) were collected with a sterile scoop. Supragingival plaque and carious tissues samples were then placed into TE buffer (PH = 7.4) with DNA free. All samples were immediately placed on dry ice and transported to a −80°C freezer for storage prior to further analysis. The six categories represent different ecological niches: SE.FMIX (*n* = 8), SE.SMIX (*n* = 8), SE.DMIX (*n* = 8), SE.S (*n* = 8), CF.FMIX (*n* = 8), CF.S (*n* = 8) (Supplementary Table 1).

### DNA Extraction and Next-Generation Sequencing

The fungal ITS2 region was amplified using PCR. The forward primer sequence (gITS7ngs) was GTGARTCATCRARTYTTTG, and the reverse primer sequence (ITS4ngs) was TCCTSCGCTTATTGATATGC (23). Total genomic DNA from samples was extracted by CTAB/SDS method, and the DNA purity and concentration were detected by agarose gel electrophoresis. An appropriate amount of the sample DNA was put into a centrifuge tube, and the sample was diluted to 1 ng/μl with sterile water. Taking the diluted genomic DNA as the template and using specific primers with barcodes, Phusion^®^ High-Fidelity PCR Master Mix with GC Buffer from New England Biolabs with GC Buffer and high-efficiency enzymes for PCR were used to ensure amplification efficiency and accuracy. PCR products were purified using GeneJET kit (Thermo Scientific). Ion Plus Fragment Library Kit 48 rxns (Thermo Scientific) was used to construct the library. The library quality was assessed after Qubit quantification and testing and then was sequenced using Ion S5™XL.

### Statistical Analysis

Using Cutadapt (Version 1.9.1) (24) to trim low-quality reads and then split the sample data, barcodes and primer sequences were cut off for preliminary quality control to obtain raw data. Read sequences were compared with species annotation database to detect chimeric sequence (25). Finally, chimeric sequences were removed to generate clean reads.

Uparse software (Version 7.0.1001) (26) was used to cluster all clean reads of all samples. Sequence clustering into operational taxonomic units (OTUs) was performed with 97% identity. Blast method (27) in QIIME software (Version 1.9.1) and Unit (Version 7.2) database (28) was used for species annotation analysis of OTUs sequences, then community composition of each sample was counted at each taxonomic level. Use the MUSCLE software (Version 3.8.31) (29) to perform fast multisequence alignment to obtain the phylogeny of all OTU sequences.

The alpha and beta diversity were analyzed using QIIME software (Version 1.9.1). Statistical differences of alpha and beta diversity were analyzed using Wilcoxon rank sum test between categories by pairwise comparison. Principle coordinates analysis (PCoA) and non-metric dimensional scaling (NMDS) diagrams were drawn using R software (Version 2.15.3). Significant differences in species abundance between categories were analyzed by LEfSe method, results were statistically significant when *P-*value <0.05.

## Results

Forty-eight clinical samples from 16 children with an average age of 54.19 ± 4.58 months, 7 males and 9 females were included in this study. The number of dmfs in SE group ranged from 8 to 16, with a mean value ± SD of 11.63 ± 2.97. No significant differences with regard to socioeconomic background, oral hygiene or eating habits between SE and CF groups, which were determined by student *t*-test of Wilcoxon-test or Fisher's exact-test (Table 1).

**Table 1:** Comparison of the socioeconomic background and behavior differences of two groups of children.

**Variable**	**CF (*n* = 8)**	**S-ECC (*n* = 8)**
**Age, mo** ^**a**^	52.25 ± 3.15	56.13 ± 5.14
**Birth weight** ^**b**^		
1.5–2.5 kg	2 (25.0)	0 (0)
>2.5 kg	6 (75.0)	8 (100)
**Gestational weeks** ^**b**^		
28–36 w	4 (50.0)	1 (12.5)
>37 w	4 (50.0)	7 (87.5)
**Delivery way** ^**b**^		
Natural labor	4 (50.0)	4 (50.0)
Cesarean delivery	4 (50.0)	4 (50.0)
**Feeding pattern within 6 months of birth** ^**b**^		
Exclusively breastfeeding	4 (50.0)	2 (25.0)
Mixed	2 (25.0)	5 (62.5)
Exclusively milk powder	2 (25.0)	1 (12.5)
**Age of abstaining from night milk** ^**b**^		
≤ 2 y	4 (50.0)	3 (37.5)
>2 y	4 (50.0)	5 (62.5)
**Frequency of brushing teeth** ^**b**^		
≤ 1 time/day	4 (50.0)	3 (37.5)
>2 time/day	4 (50.0)	5 (62.5)
**Frequency of eating sweets** ^**b**^		
1–3 time/week	7 (87.5)	6 (75.0)
>3 time/week	1(12.5)	2(25.0)
**Household income per capita** ^**b**^		
<5,000 RMB	4 (50.0)	3 (37.5)
≥5,000 RMB	4 (50.0)	5 (62.5)

*Values are presented as mean ± SD or n (%)*.

*CF, caries free; S-ECC, severe early childhood caries*.

a *By student t-test of Wilcoxon test*.

b *By Fisher's exact test*.

*All comparisons are not significant (P > 0.05)*.

### Sequencing Information

Ion S5™XL sequencing generated an average of 75,951 reads per sample after quality control. With 97% identity, these sequences were clustered into 2,074 OTUs. A total of 10 fungal phyla and 281 species were observed. The rarefaction curve reflecting the amount of sequencing data and the rank abundance curve reflecting the species richness were relatively flat, indicating that the sampling was sufficient (Supplementary Figures 1, 2).

### Fungal Community Diversity and Distribution

**Figure 1:**
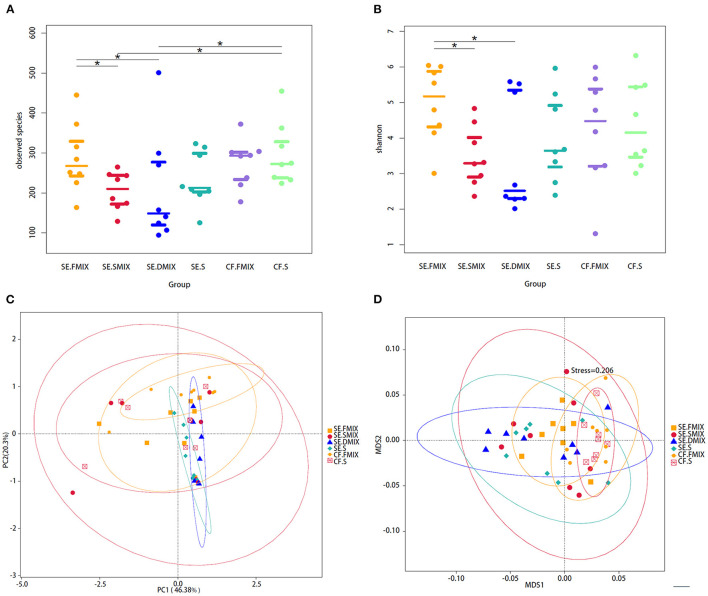
Alpha and beta diversity analyses among the six categories. **(A)** Observed species and **(B)** Shannon diversity in various spatial niches with and without S-ECC. ^*^Represents significant differences (*P* < 0.05) between two categories. **(C)** Principal coordinate analysis and **(D)** non-metric multidimensional scaling plot based on taxa abundance in each sample. Each point in figures represents a sample and samples of the same category are represented by the same color.

The diversity of fungal communities were significantly different between SE.FMIX-SE.SMIX, SE.DMIX-SE.FMIX (*P* < 0.05; Figures 1A,B). In general, the alpha diversity of healthy dental plaque communities was higher than that of plaque on the decayed teeth surfaces and carious lesion communities. There were no significant dissimilarities between saliva and plaque in either CF or SE group.

In addition, PC_O_A based on Weighted Unifrac distances and NMDS based on Bray-Curtis were used to describe the fungal community distribution of each category. The CF.FMIX communities clustered separately from SE.FMIX [multiple response permutation procedure (MRPP): *P* < 0.05, *A* = 0.03]. Obvious differences of CF.S communities were also observed when compared with the SE.S (MRPP: *P* < 0.01, *A* = 0.05) (Figures 1C,D; Supplementary Table 2).

### Fungal Community Composition

**Figure 2:**
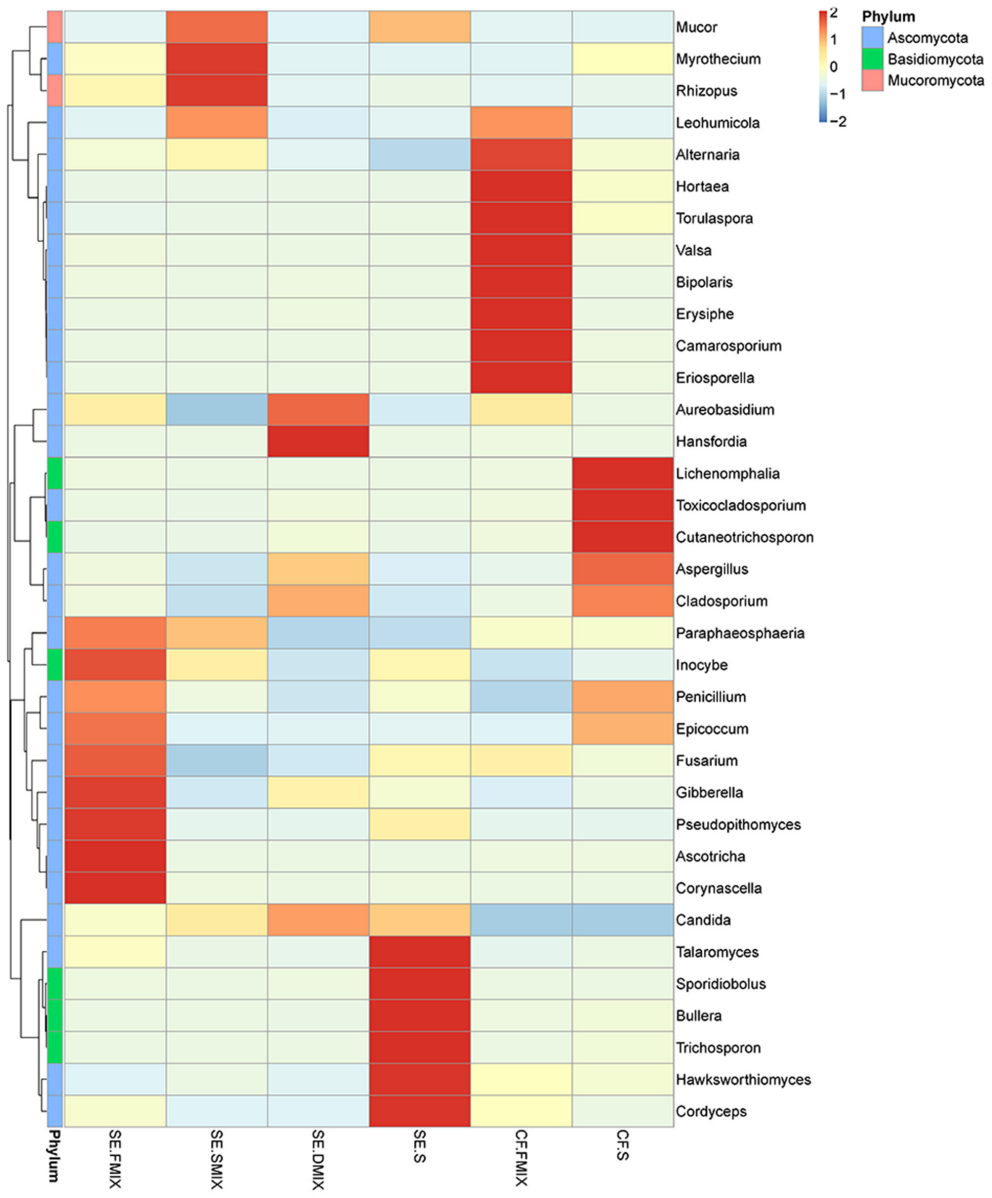
Heat map of the 35 most abundant genera for each category. Sample information was in the vertical direction, annotation information was in the horizontal direction.

**Figure 3:**
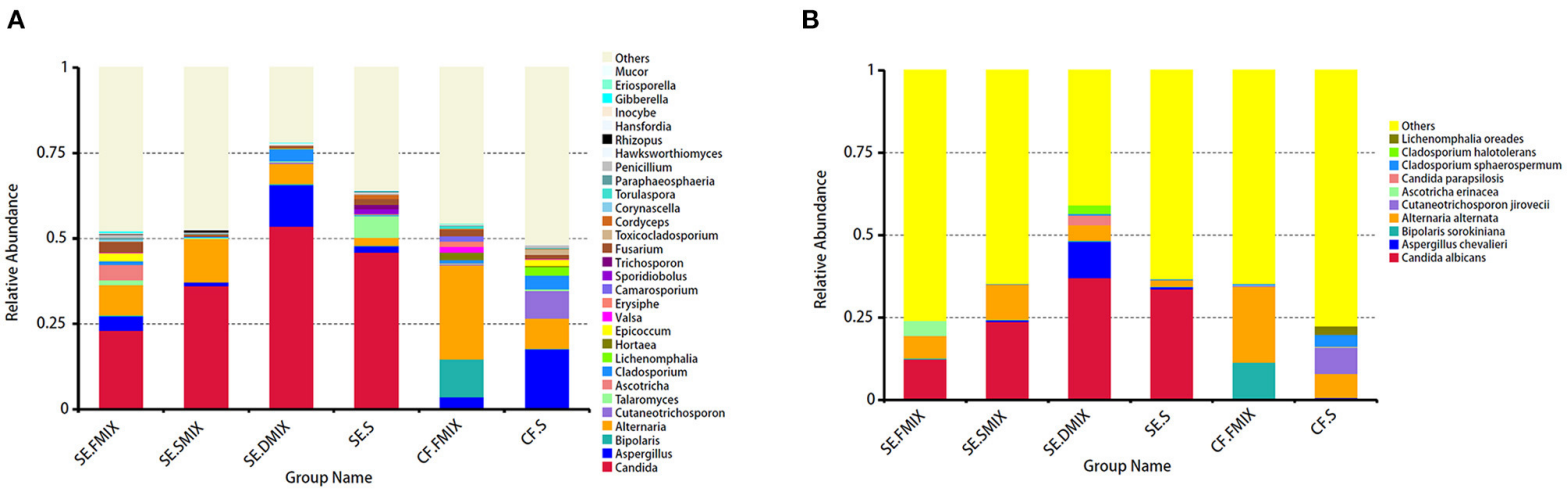
Distribution of fungal profiles in children with S-ECC and caries-free. **(A)** At the genus level, top 30 taxa for each category. **(B)** Top 10 fungal species in the six categories.

To investigate the taxonomic differences in the six categories, the top 35 most abundant genera were selected for cluster analyses. Heat map showed that the composition of the microbial community varied greatly among the six categories (Figure 2). The most abundant phyla were *Ascomycota* (SE.FMIX, 59.2%; SE.SMIX, 56.9%; SE.DMIX, 80.0%; SE.S, 65.2%; CF.PF, 60.4%; CF.S, 43.1%) and *Basidiomycota* (SE.FMIX, 1.5%; SE.SMIX, 0.5%; SE.DMIX, 1.1%; SE.S, 6.0%; CF.PF, 1.1%; CF.S, 12.8%) (Supplementary Figure 3). Dominant genera were *Candida* (26.7%), *Aspergillus* (6.6%), *Bipolaris* (2.0%) *Alternaria* (11.0%), *Cutaneotrichosporon* (1.5%), *Talaromyces* (1.5%), *Cladosporium* (1.7%), and *Fusarium* (1.6%) regardless of caries status. *Aspergillus, Alternaria, Talaromyces, Cladosporium*, and *Fusarium* were present in all samples (Figure 3A; Table 2). Community composition of dominant genera across the six categories described in Supplementary Table 3.

**Table 2:** Predominant genera in all samples (mean relative abundance > 1%).

**OTU**	**Fungal taxa**	**% Mean relative**	**% Detection**
**number**		**abundance ± SD**	**rate**
OTU_1	*Candida*	26.7 ± 40.5	97.9
OTU_6	*Aspergillus*	6.6 ± 17.2	100
OTU_3	*Bipolaris*	2.0 ± 12.3	70.8
OTU_2	*Alternaria*	11.0 ± 20.6	100
OTU_5	*Cutaneotrichosporon*	1.5 ± 9.2	72.9
OTU_9	*Talaromyces*	1.5 ± 6.8	100
OTU_21	*Cladosporium*	1.7 ± 4.9	100
OTU_23	*Fusarium*	1.6 ± 2.2	100

At species level, *C. albicans* was the most abundant taxon among all the categories of SE group (SE.FMIX:12.5%; SE.SMIX:23.9%; SE.DMIX:37.2%; SE.S:33.7%), while the most important fungal species in CF group were *Alternaria alternata* (CF.FMIX, 23.0%; CF.S, 7.3%) (Figure 3B).

### Differential Species Analyses Among Categories

**Figure 4:**
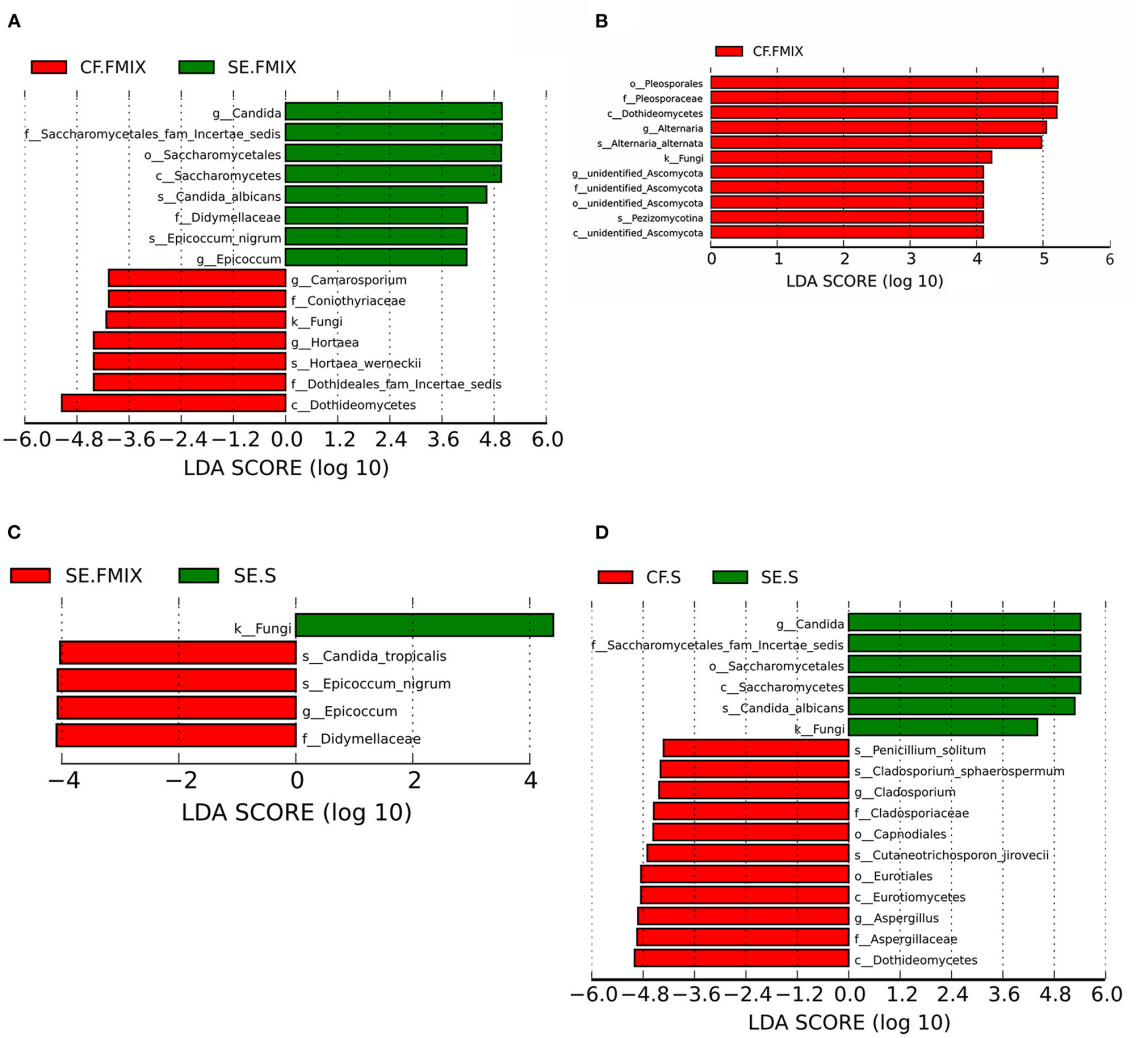
Differential species among categories (Defined by LEfSe). The histograms show species with LDA scores greater than 3. **(A)** Differential species between CF.FMIX and SE.FMIX, **(B)** CF.FMIX and CF.S, **(C)** SE.FMIX and SE.S, and **(D)** CF.S and SE.S.

Differential species in various ecological niches in CF and SE groups were detected by LEfSe. The abundance of *Hortaea werneckii* in CF.FMIX was significantly higher than that in SE. FMIX, while *C. albicans* and *Epicoccum nigrum* remarkably enriched in SE. FMIX (Figure 4A; Supplementary Figure 4; *P* < 0.05). Compared to CF.S, *Alternaria alternata* and *Pezizomycotina* were more abundant in CF.FMIX (Figure 4B; Supplementary Figure 5; *P* < 0.05). For samples collected from SE.FMIX and SE.S, significant differences in fungal taxa were observed, with *Candida tropicalis* and *Epicoccum nigrum* more abundant in SE.FMIX category (Figure 4C; Supplementary Figure 6; *P* < 0.05). *Cutaneotrichosporon jirovecii, Cladosporium sphaerospermum* and *Penicillium solitum* were differentially abundant between CF.S and SE.S categories (Figure 4D; Supplementary Figure 7; *P* < 0.05).

## Discussion

In this study, we investigated the fungal profiles in saliva and different spatial niches of primary teeth in S-ECC and caries-free children by amplifying fungal ITS2 region, which provided the basis for a comprehensive understanding of oral fungal microecology. Ten fungal phyla were identified, while previous studies have reported two or three fungal phyla (10). This inconsistency may be due to our multi-space sampling, sequencing technology and population.

Higher fungal community diversity and richness in the SE. FMIX category than in SE. SMIX and SE. DMIX categories, indicating that the low alpha diversity of mycobiome may be associated with caries. Jesus and Connell et al. also reported high alpha diversity in healthy supragingival plaques (12, 18). Moreover, our previous longitudinal study focusing on oral microflora of children with deciduous teeth showed that low microbial diversity was related to the occurrence of S-ECC (30). The alteration of oral community diversity (bacteria and fungi) may be caused by the changes in local acidic environment. Scientists believe that the accumulation of lactic acid results in a decrease of local pH, some microorganisms are eliminated by low pH, and acid-producing and acid-tolerant taxa survived (31, 32). However, supragingival plaque analyses of 17 children aged 7–10 years by Fechney et al. showed no significant differences in richness and evenness between caries and healthy dentitions (10). Currently, mostly cross-sectional studies have been used to investigate oral fungal communities. Longitudinal studies will be necessary in the future to determine the causal relationship between alpha diversity and the occurrence and development of dental caries.

Beta diversity indices demonstrated that the fungal composition was quite different between CF.S and SE.S groups. Studies have demonstrated that in a healthy state, various microorganisms in the oral cavity maintain the balance of the microbial community through antagonism, symbiosis, and competition (31, 33). It is widely believed that changes of major environmental factors (such as excessive dietary carbohydrate intake) will facilitate the overgrowth of disease-related taxa and transform the microbial community into a pathological state (6, 34, 35), which ultimately leads to dental caries. Our results support a clear separation of oral fungal communities in healthy and S-ECC children, indicating that the prevalence of S-ECC has disrupted the unique microecological stability and affected the environment homeostasis of oral microecological system. In addition, we did not detect remarkable taxonomic community dissimilarities between SE. SMIX and SE. DMIX, which implied that the microbial community might tend to stabilize once the caries environment is established.

Our results suggested that SE.FMIX had obvious taxa clustering distinction compared to CF.FMIX (*P* < 0.05), yet was more similar to SE. SMIX community composition. This result indicated that the healthy teeth in S-ECC children also have an excessive danger of developing caries. Similarly, an article published in 2020 on site-specific analyses of the fungal community showed that compared with those of supragingival plaque community on the healthy teeth surface in CF group, fungal profiles of the healthy teeth surface plaque from children with dental caries was more similar to those of the plaque of decayed teeth (12). Oral epidemiological studies have indicated that children who experienced primary dental caries had a much higher risk of developing caries than those who are healthy (36, 37). Therefore, it is necessary to apply some clinical preventive measures on the healthy teeth of children with dental caries to reduce the possibility of suffering dental caries.

We pioneered a fungal community comparison of supragingival plaque and saliva from children with and without S-ECC. No remarkable fungal community dissimilarities between saliva and plaque were observed in either CF or S-ECC group. The possible explanation was that microbes attached to the surfaces of the teeth are constantly flowing into the saliva (38, 39), making the saliva communities more similar to supragingival plaque communities. However, healthy teeth plaque was colonized with unique fungi compared with saliva, including *Alternaria alternata* and *Pezizomycotina* in the CF group and *Candida tropicalis* and *Epicoccum nigrum* in the SE group.

In present study, *C. albicans* was the most abundant species in S-ECC children, and was present in 94% of the samples. Currently, *C. albicans* is regarded as a caries-related fungus and is the most widely studied fungal species (12, 17, 40). Epidemiological investigations have shown that children with *C. albicans* had a higher risk of suffering caries than children who did not (17, 41). *C. albicans* is an acidogenic and acid-resistant species with independent cariogenic ability. It can invade dentin tubules, bind to denatured collagen, and secrete aspartyl protease, leading to the disintegration and dissolution of tooth hard tissues (42–45). Moreover, coculture of *C. albicans* with the cariogenic bacteria *S. mutans* has been proven to increase the amount of extracellular polysaccharides (EPS) (40, 46), *C. albicans* colonization can enhance the acid-producing capacity of polymicrobial biofilms, induce the microbial dysbiosis (47), and consequently prompt tooth demineralization. However, some researchers believe that *C. albicans* are frequent in all individuals with and without dental caries. Consequently, the role of *C. albicans* in the occurrence of caries has not been recognized (10, 18, 48). Culture-based methods have detected *Candida* (*C. albicans* and *Candida dubliniensis*) exist in carious lesions (49, 50). In the present study, the abundance of *C. albicans* in SE.FMIX (12.5%), SE.SMIX (24.0%), and SE.DMIX (37.2%) increased successively, indicating that *C. albicans* plays an increasingly important role in deepening dental caries.

Currently, few studies have explored the fungal community diversity in carious lesions. To detect the mycobiome in carious tissues, we used the next generation sequencing (NGS) technology and found that the dominant genera included *Candida* (53.8%), *Aspergillus* (12.1%), *Alternaria* (5.8%), and *Cladosporium* (3.5%). Our results implied that fungal taxonomic diversity also exists in carious lesions. Previous studies have usually focused on bacterial diversity in carious lesions. Researchers have found that carious lesions contain considerable microorganisms (51, 52). Due to their specific location and relatively closed environment, the carious tissues may have a unique microbial community and may contribute to the development of anaerobic conditions (53). We hypothesized that the similar changes might occur in the fungal community of carious lesions, which needed to be verified in future laboratory studies. In addition, this is a preliminary study with limited sample size. It is meaningful to conduct a longitudinal study with a larger sample size to explore the temporal and spatial succession of oral fungal community composition and diversity in children.

## Conclusions

In conclusion, the diversity of fungal communities in S-ECC children showed significant differences in various spatial niches of primary teeth. The successively increasing abundances of *C. albicans* in SE.FMIX, SE.SMIX and SE.DMIX indicated that *C. albicans* richness was closely related to the caries states and depth. Salivary and plaque community compositions were similar in either CF or SE group. However, there were still specific species colonizing in different oral spatial niches.

## Data Availability Statement

The datasets presented in this study can be found in online repositories. The names of the repository/repositories and accession number(s) can be found below: https://www.ncbi.nlm.nih.gov/bioproject/PRJNA753287, PRJNA753287.

## Ethics Statement

The studies involving human participants were reviewed and approved by Ethics Committee of the Hospital of Stomatology, Sun Yat-sen University, in Guangzhou, China. Written informed consent to participate in this study was provided by the participants' legal guardian/next of kin.

## Author Contributions

YC, YT, and HL participated in the study design. YC, YW, and YZha performed the data analysis and interpretation. YC, YZho, LP, YW, YZha, and YT participated the sample collections. YC wrote the paper. All authors strictly revised the important contents of the manuscript and approved the final version.

## Funding

This study was funded by the National Natural Science Foundation of China (Grant No. 31901116).

## Conflict of Interest

The authors declare that the research was conducted in the absence of any commercial or financial relationships that could be construed as a potential conflict of interest.

## Publisher's Note

All claims expressed in this article are solely those of the authors and do not necessarily represent those of their affiliated organizations, or those of the publisher, the editors and the reviewers. Any product that may be evaluated in this article, or claim that may be made by its manufacturer, is not guaranteed or endorsed by the publisher.
