# Supplementary material for: Oral Mycobiome Differences in Various Spatial Niches With and Without Severe Early Childhood Caries
Source: Front Pediatr. 2021 Nov 15;9:748656. doi: 10.3389/fped.2021.748656 (PMC8634708; doi:10.3389/fped.2021.748656)
Supplement: Supplementary file 1 [file Data_Sheet_1.PDF]

Supplementary Figures

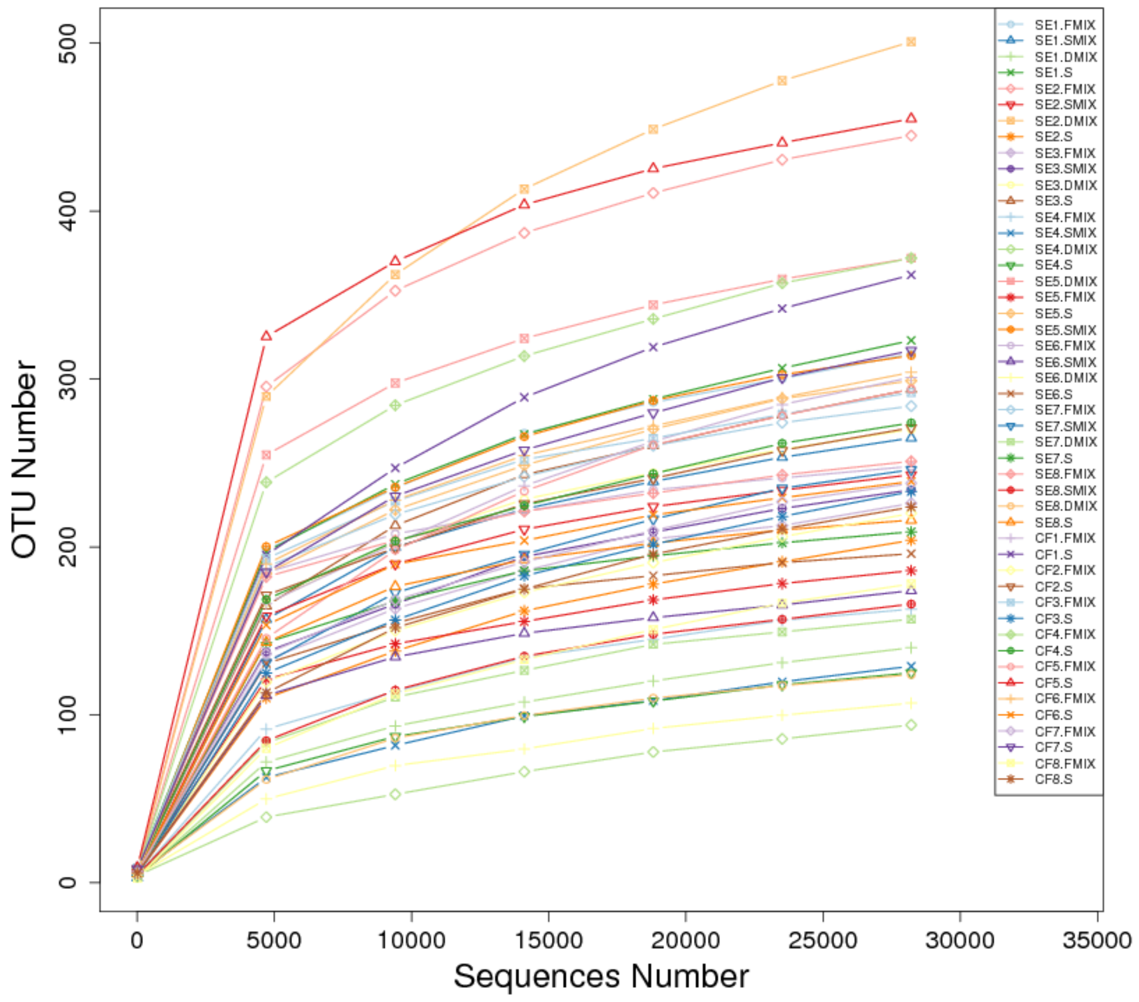

Supplementary Figure 1. Rarefaction curve of the 48 samples. The abscissa is the number of sequences randomly selected from a sample, and the ordinate is the OTU counts that can be constructed based on the number of sequencing pieces, which is used to reflect the depth of sequencing.

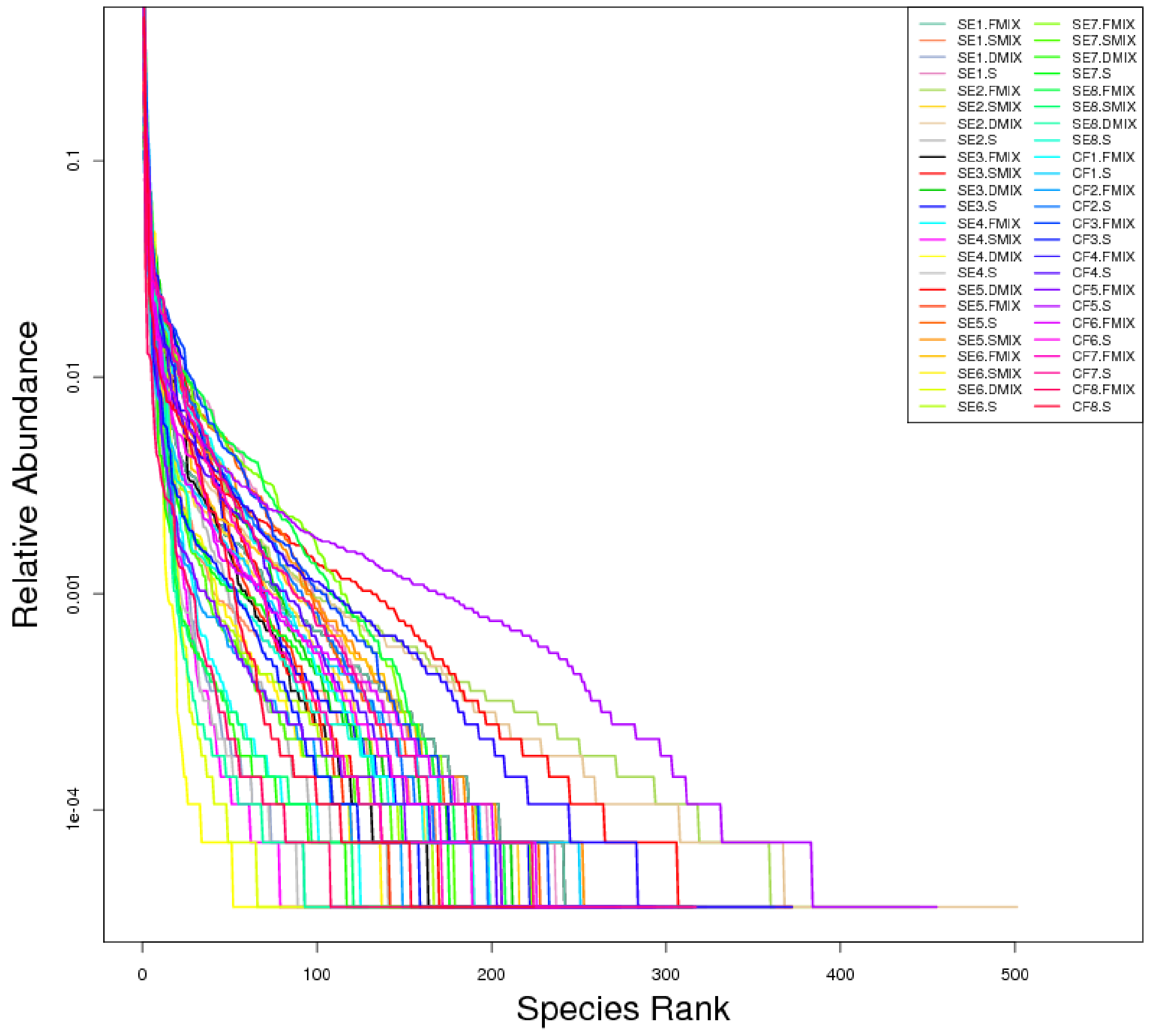

Supplementary Figure 2. Rank abundance of the 48 samples. The abscissa is the sequence number sorted by the abundance of OTUs, the ordinate is the relative abundance of corresponding OTUs.

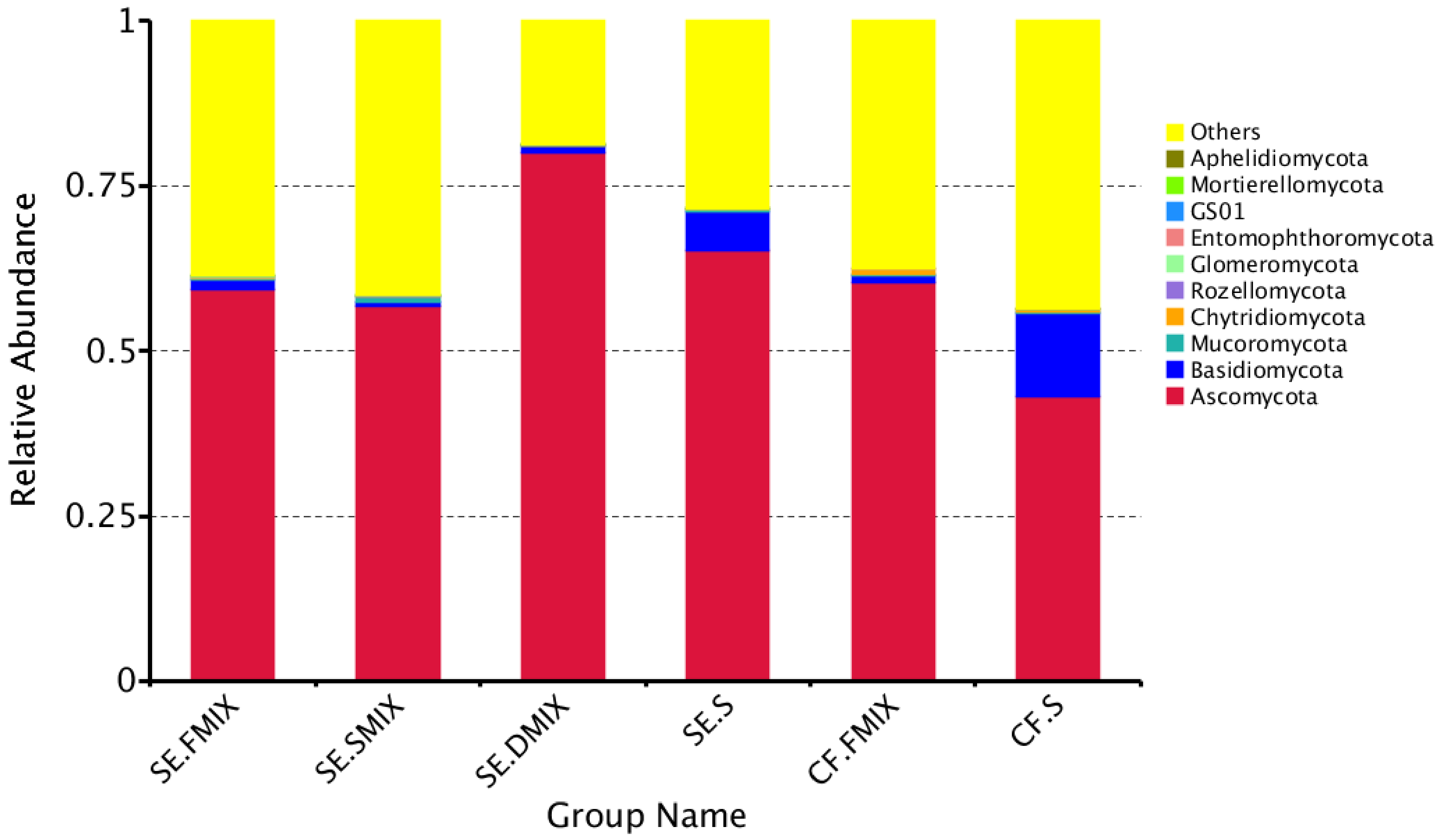

Supplementary Figure 3. At the phyla level, distribution of fungal profiles in children with S-ECC and caries-free for each category.

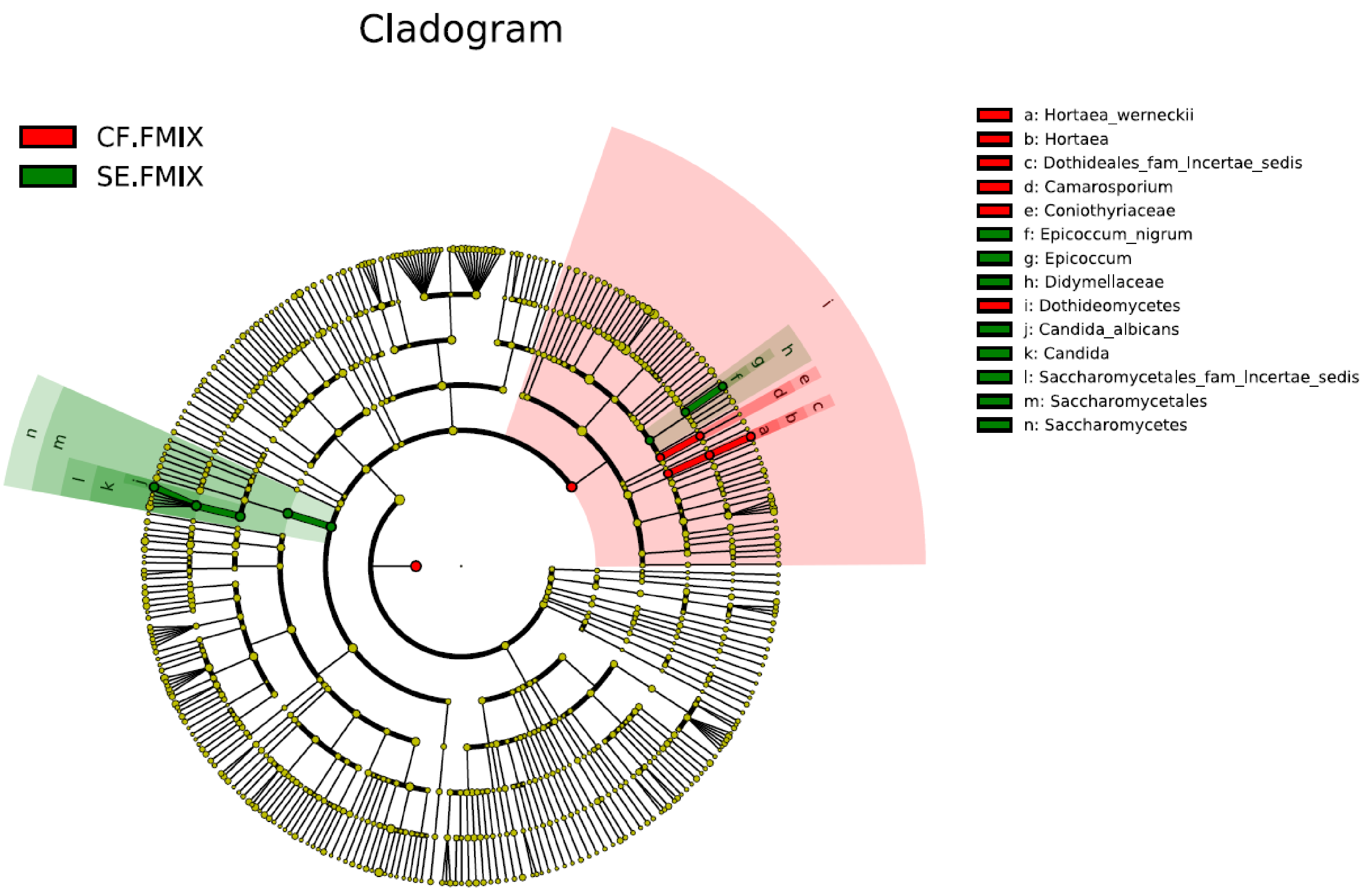

Supplementary Figure 4. Cladogram for taxonomic representation of significant differences between CF.FMIX and SE.FMIX.

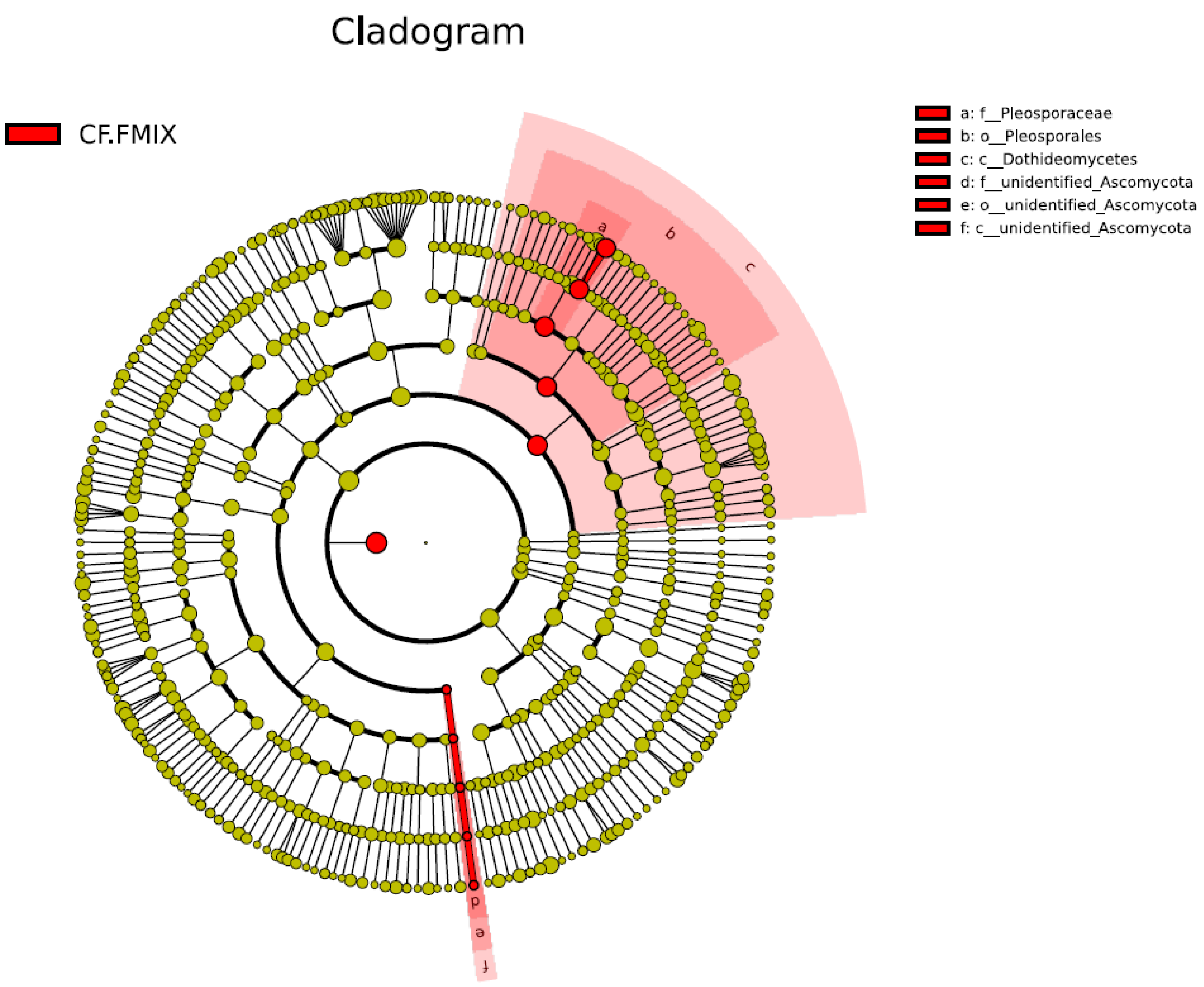

Supplementary Figure 5. Cladogram for taxonomic representation of significant differences between CF.FMIX and CF.S.

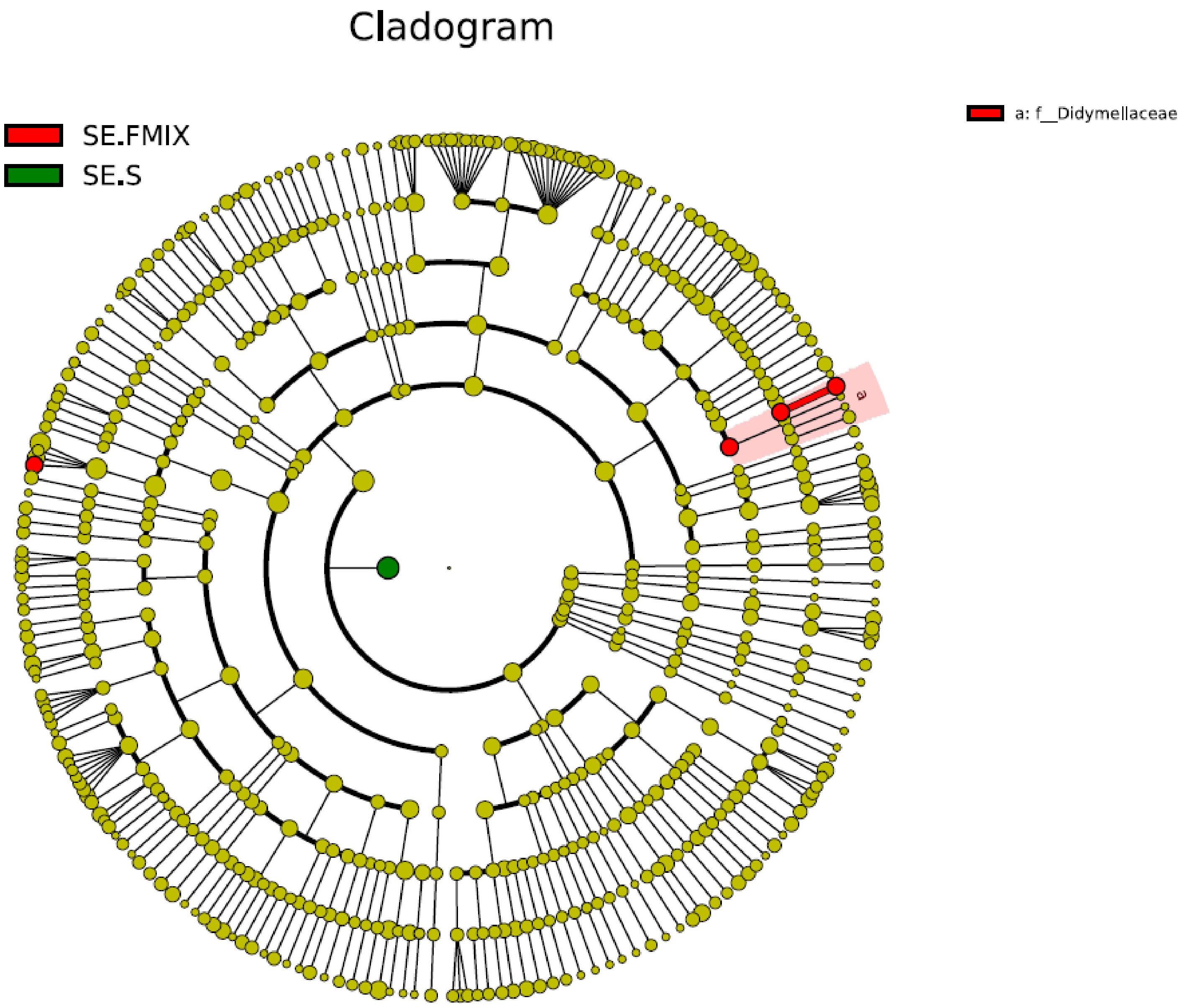

Supplementary Figure 6. Cladogram for taxonomic representation of significant differences between SE.FMIX and SE.S.

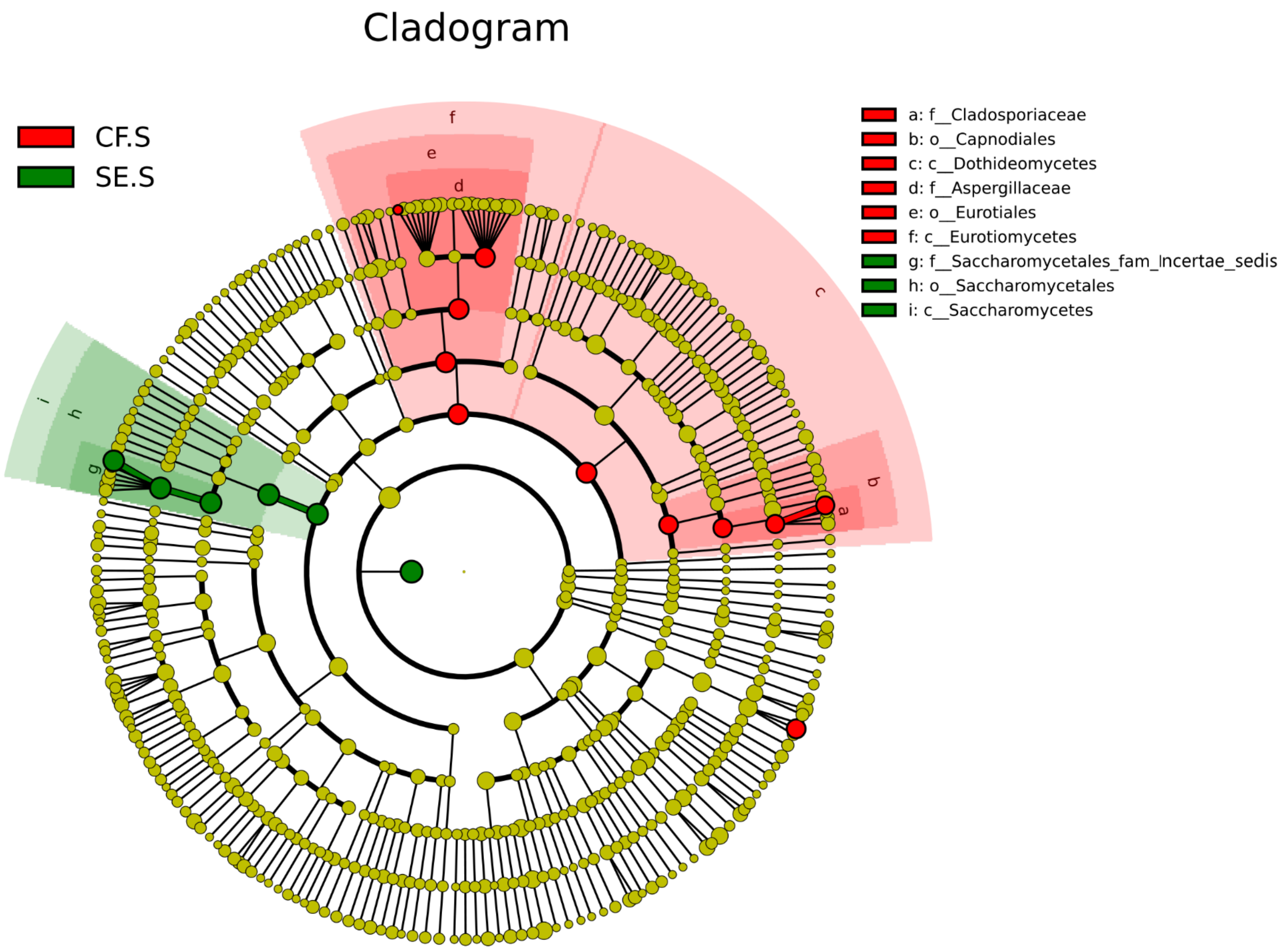

Supplementary Figure 7. Cladogram for taxonomic representation of significant differences between CF.S and SE.S.
